# Effects of Transcription-Dependent Physical Perturbations on the Chromosome Dynamics in Living Cells

**DOI:** 10.3389/fcell.2022.822026

**Published:** 2022-07-07

**Authors:** Hyeyeong Ku, Gunhee Park, Jiyoung Goo, Jeongmin Lee, Tae Lim Park, Hwanyong Shim, Jeong Hee Kim, Won-Ki Cho, Cherlhyun Jeong

**Affiliations:** ^1^ Chemical and Biological Integrative Research Center, Korea Institute of Science and Technology (KIST), Seoul, South Korea; ^2^ KHU-KIST Department of Converging Science and Technology, Kyung Hee University, Seoul, South Korea; ^3^ Department of Biological Sciences, Korea Advanced Institute of Science and Technology (KAIST), Daejeon, South Korea; ^4^ Division of Life Sciences, Korea University, Seoul, South Korea; ^5^ Department of Oral Biochemistry and Molecular Biology, Kyung Hee University, Seoul, South Korea; ^6^ KI for Health Science and Technology (KIHST), Korea Advanced Institute of Science and Technology (KAIST), Daejeon, South Korea

**Keywords:** chromatin dynamics, CRISPR labeling, telomere, single-particle tracking, transcription, gene expression regulation, epigenetic modulation, fluorescence microscopy

## Abstract

Recent studies with single-particle tracking in live cells have revealed that chromatin dynamics are directly affected by transcription. However, how transcription alters the chromatin movements followed by changes in the physical properties of chromatin has not been elucidated. Here, we measured diffusion characteristics of chromatin by targeting telomeric DNA repeats with CRISPR-labeling. We found that transcription inhibitors that directly block transcription factors globally increased the movements of chromatin, while the other inhibitor that blocks transcription by DNA intercalating showed an opposite effect. We hypothesized that the increased mobility of chromatin by transcription inhibition and the decreased chromatin movement by a DNA intercalating inhibitor is due to alterations in chromatin rigidity. We also tested how volume confinement of nuclear space affects chromatin movements. We observed decreased chromatin movements under osmotic pressure and with overexpressed chromatin architectural proteins that compact chromatin.

## Introduction

The molecular genetic material, DNA, exists in a form of chromatin in eukaryotes throughout most of the cell cycle. Chromatin is a higher-order fiber structure of DNA formed by nucleosomes composed of histones. It is located inside a nucleus separated from cytoplasm by a physical barrier, known as a nuclear membrane. Transcription and many other gene regulatory processes occur within the chromatin context in the compartmentalized nuclear space.

Recently established chromatin conformation capture (3C)-based sequencing and advanced live-cell imaging techniques have suggested that cell-type specific intrinsic structures and dynamics of chromatin are responsible for regulating gene expression (Rowley and Corces, 2018; Oudelaar and Higgs, 2021). A series of studies using 3C-based sequencing have shown that chromatin sub-organization reflects different gene expression patterns across cell types (Dekker and Mirny, 2016). Moreover, chromatin is folded into unique loop structures, so-called topologically associated domains (TADs), by chromatin architectural proteins including CTCF and Cohesin complex. These TADs provide spatial constraints that promote physical contacts among gene regulatory elements within a single TAD (Dixon et al., 2012; Nora et al., 2012; Rao et al., 2014; Chang et al., 2020).

Accumulated evidence with real-time tracking of chromatin loci in live cells indicates that dynamics of chromatin are related to gene expression regulation, rather than mere thermal motions in the cell nucleus (Babokhov et al., 2020). Given the fact that molecular components for transcription, such as RNA polymerases, transcription factors, and the chromatin architectural proteins along with chromatin fibers, are physical entities with masses and volumes, the mobility of chromatin should be altered by their interactions for transcriptional controls.

Here, we investigated how alterations in physical properties of chromatin, rigidity, and volume confinement, influence chromosome dynamics in human cells. We found chromosome movements are directly affected by changes in physical properties of chromatin by calculating diffusion constants with single locus tracking in live-cell nuclei. Because the movement of chromosomes is heterogenous in the living cell nucleus, we statistically measured the motions of each chromosome by labeling telomere loci with a CRISPR/dCas9-based labeling system (Hilbert et al., 2021).

## Results

### Fluorescently Labeling Chromosome *via* Telomeres for Single Locus Tracking

**FIGURE 1:**
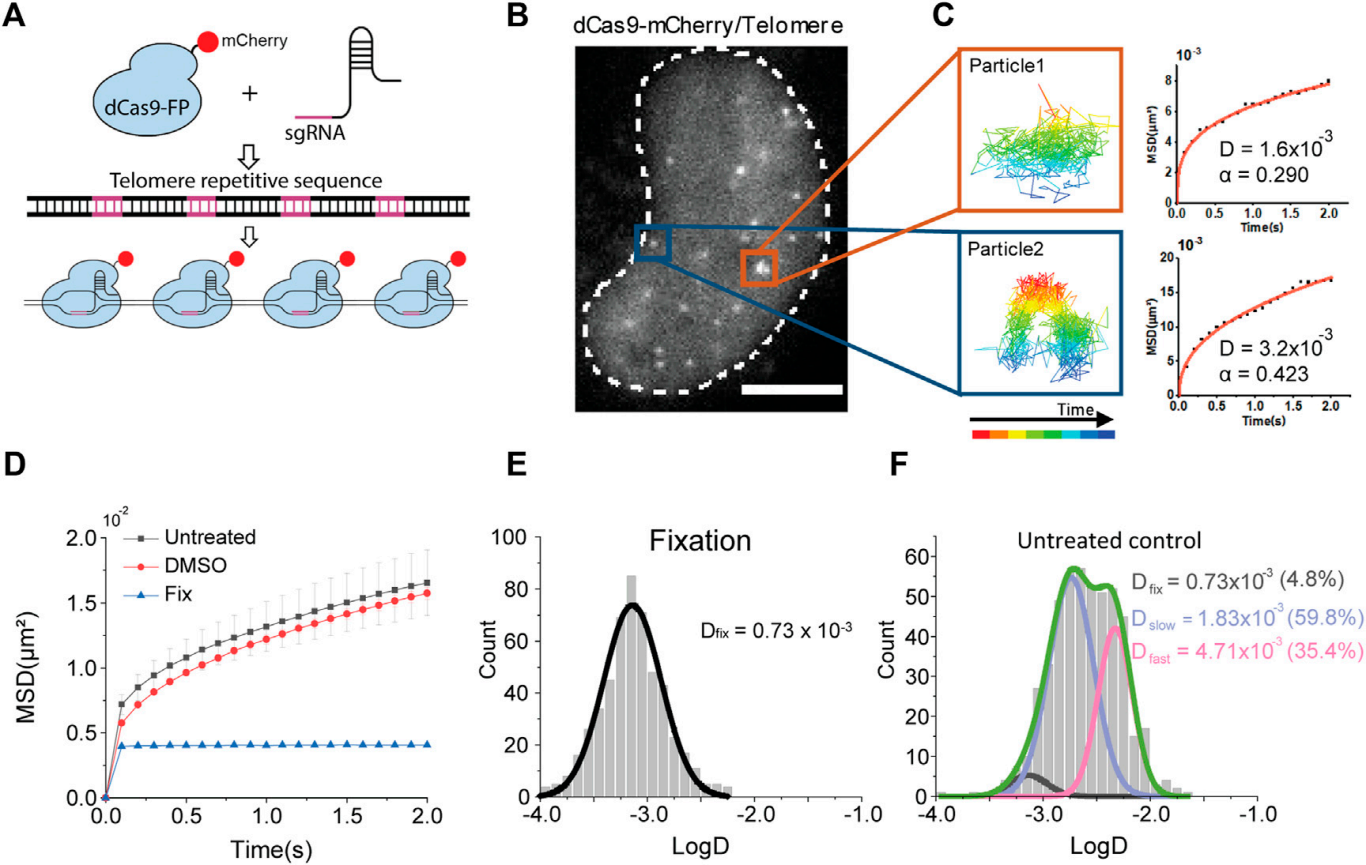
Single chromatin locus tracking and diffusion analysis. **(A)** A schematic representation of CRISPR/dCas9-labeling on telomere sequences. **(B)** A representative image of fluorescently labeled chromatin loci in a living cell nucleus. The scale bar represents 5 μm. **(C)** Examples of single locus tracking for diffusion analysis. **(D)** Averaged MSD-t plots for each time interval in live cells under normal culture media (N = 34 cells, *n* = 520 trajectories), DMSO treatment (N = 32 cells, *n* = 653 trajectories), and fixed cells (N = 32 cells, *n* = 575 trajectories). **(E,F)** Distributions of diffusion constants for individual trajectories in **(E)** fixed cells and **(F)** normally cultured cells.

To label specific chromatin loci within a single cell nucleus, we adopted CRISPR/dCas9-based labeling system which allows us to stably label specific DNA sequences by using nuclease-deactivated Cas9 (dCas9) with a small guide (sg) RNA (Chen et al., 2013; Gu et al., 2018). To visualize DNA regions, dCas9 fused with fluorescent protein is guided to the specific sites by sgRNAs which often target multiple nearby sequences to achieve a signal-to-noise ratio above a background fluorescence intensity. However, there is a limitation that a robust way to express multiple sgRNAs in the cell has not been established. Although a method to insert multiple sgRNAs in a single expression vector has recently been introduced, it requires a thorough optimization process to validate functional sgRNAs in the vector (Gu et al., 2018). Hence, one convenient way to label chromatin is by targeting telomere sequences which consist of repeated sequences conserved across different chromosomes (Moyzis et al., 1988). By targeting telomeres, we could label multiple genomic sites with a single sgRNA in the cell nucleus (Figure 1A).

We expressed the sgRNA targeting human telomere regions along with a fluorescent protein, mCherry, fused dCas9 in HeLa cells (Figure 1B). Fluorescence imaging with highly inclined and laminated optical (HILO) sheet illumination could capture telomere loci in the nucleus. We continuously imaged the telomere loci for 2 minutes and analyzed each trajectory of the locus to calculate diffusion constants (Figure 1C). We found most of the chromatin movements show confined diffusions.

### Diffusion Analysis of Chromosome Movement

We plotted averaged mean square displacement (MSD) over time from trajectories for diffusion analysis -is normally cultured Hela cells (Figure 1D). We note that we collected more than 200 trajectories from at least 20 cells for each experimental condition throughout this study to secure statistical significance.

Since chromatin is a relatively long polymer within a confined space, we fitted the MSD-t plot with the anomalous diffusion equation, 4*Dt* ^α^, for two-dimensional motions. We found an apparent diffusion constant from all over the trajectories, *D*_app_ was 3.33 (±0.001, s. e.) x 10^–3^ μm^2^/s with an anomalous coefficient *α* = 0.292 (±0.004, s. e.) (Supplementary Figure S1, Table S1). We could not observe any significant changes under dimethyl sulfoxide (DMSO), a solvent for reagents in the following experiments. We next analyzed fluctuations of the chromatin loci in fixed cells to check the thermal diffusion constant, which is measured as 0.101 (N.S.) × 10^−3^ μm^2^/s. We performed fixed cell imaging under the same condition used for live cell imaging.

A distribution of *D*_app_ from individual trajectories under the normal condition, however, showed a multimodal distribution compared to a distribution from fixed cells (Figures 1E,F; Table S2). We fitted the distribution with triple Gaussian peaks, assuming that the multimodal distribution of *D*_app_ is due to mixed trajectories from 1) a global long-range movement of chromatin (*D*_fast_), 2) a local movement of chromatin loci (*D*_slow_), and 3) an immobilized portion with thermal fluctuation under the imaging condition (*D*_fixed_). (Chen et al., 2013; Gu et al., 2018). For fitting with triple Gaussian peaks, we fixed the diffusion constant of thermal fluctuation as *D*_fix_ = 0.73 (±0.06, s. e.) × 10^−3^ μm^2^/s obtained from fixed cells, since we performed all imaging under the same condition (Figure 1F).

### Effects of Transcriptional Perturbations on Chromosome Movement

We next sought to test the effects of transcription activity on chromatin dynamics with known transcription inhibitors; triptolide, 5,6-Dichloro-1-β-d-ribofuranosyl-benzimidazole (DRB), flavopiridol, alpha-amanitin, and actinomycin D, which act at different steps during transcription. Triptolide is an inhibitor for blocking transcription initiation, which covalently binds to X-box binding protein 1 (XBP1), one of the two helicase subunits of an initiation transcription factor Transcription factor II Human (TFIIH) (Titov et al., 2011). Flavopiridol and DRB inhibit transcription at the elongation step by inhibition of positive-transcription elongation factor b (P-TEFb) (Chao and Price, 2001; Peterlin and Price, 2006). Alpha-amanitin and actinomycin D are global transcription inhibitors by specifically binding to a catalytic active site of RNA polymerase II and intercalating into DNA at GpC sites, respectively (Kirk, 1960; Jacob et al., 1970).

**FIGURE 2:**
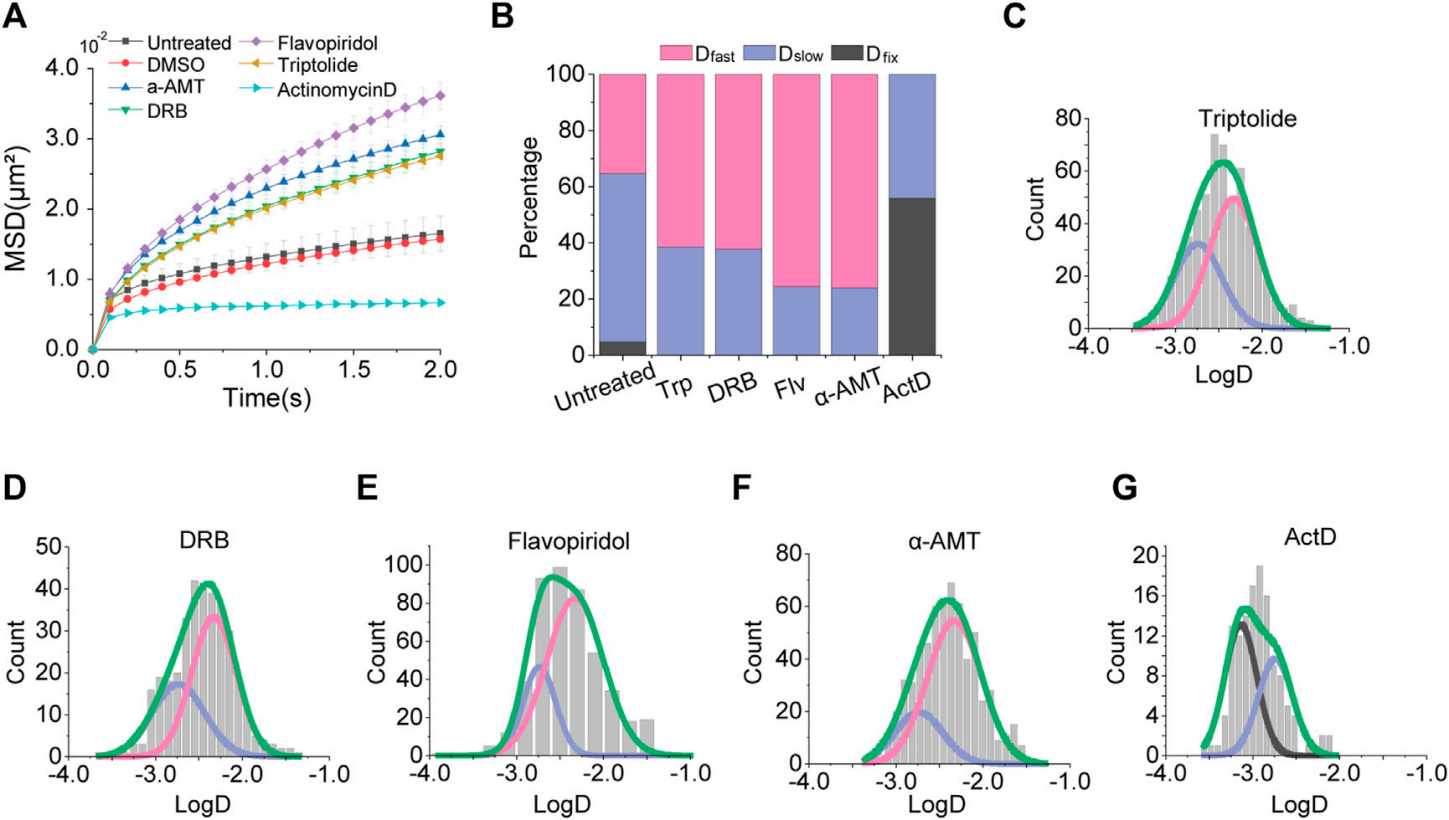
Effects of transcription inhibitors. **(A)** Averaged MSD-t plots for each time interval is normally cultured cells and transcription inhibitor treated cells. (triptolide, N = 27 cells, *n* = 633 trajectories; DRB, N = 22 cells, *n* = 370 trajectories; flavopiridol, N = 30 cells, *n* = 501 trajectories; a-amanitin, N = 33 cells, *n* = 671 trajectories; actinomycin D, N = 33 cells, *n* = 633 trajectories) **(B)** Percentages of diffusion constant components from multimodal Gaussian fittings for each condition. **(C–G)** Distributions of diffusion constants for individual trajectories in cells treated with triptolide, DRB, flavopiridol, a-amanitin, and actinomycin D, respectively. Green lines represent fitting with triple Gaussian peaks for the entire distribution. Pink, blue, and black lines represent single Gaussian portions for fast, slow, and fixed diffusions, respectively.

Under treatment of each transcription inhibitor, we measured the apparent diffusion constant (Figure 2A). Interestingly, we observed a consistent increase in chromatin mobility for most transcription inhibitors we used, except for actinomycin D. We confirmed that the effect of a transcription inhibitor is general in another cell line, HEK293 (Supplementary Figure S2). The MSD-t plot for actinomycin D treatment showed a drastic decrease in the chromatin movements. These results imply that transcription restrains the mobility of chromatin. However, the inconsistent results from actinomycin D treatment may be due to the difference mechanisms for transcription inhibition because actinomycin D intercalates into DNA all over the chromatin, unlike other transcription inhibitors which merely target specific transcription factors.

We further checked multimodal distributions of diffusion constants for each inhibitor and represented the results of multiple Gaussian fitting as bar graphs for comparison (Figure 2B). We fitted the distributions with a triple Gaussian. However, if triple Gaussian fitting is failed, we fitted with double Gaussian without a peak failed for triple Gaussian peaks (Figures 2D–G). For example, a fitting with triple Gaussian peaks for DRB treatment failed for *D*_fixed_, then we fitted the distribution with double Gaussian peaks without the *D*_fixed_ peak (Supplementary Figure S3).

We found a consistent increase in a portion of *D*_fast_ and a decrease in a portion of *D*_slow_ for inhibitors that target transcription factors (Figures 2B–G). These results indicate that the inhibition of transcription promotes global movements of chromatin by removing domestic constraints, such as the disassociation of transcription factors from chromatin so that it gives more flexibility to chromatin. Under these conditions, the *D*_fixed_ portion disappeared.

On the other hand, the treatment of actinomycin D showed a completely different distribution (Figures 2B,G). Under actinomycin D treatment, the *D*_fast_ portion disappeared, while *D*_fixed_ was dominantly increased. We speculate this result may be due to an increase in chromatin rigidity induced by the effect of DNA intercalation.

### Effects of DNA Intercalators on Chromosome Movement

**FIGURE 3:**
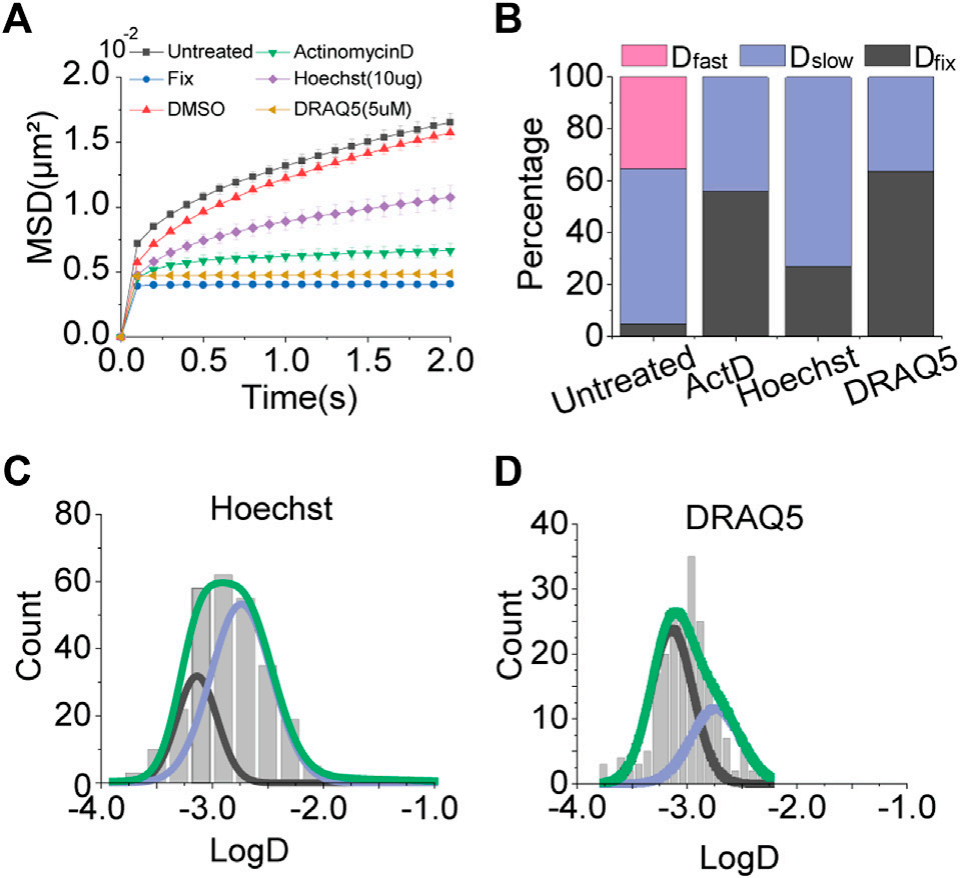
Effects of DNA intercalators. **(A)** Averaged MSD-t plots for each time interval is normally cultured cells and DNA intercalator treated cells. (Hoechst, N = 20 cells, n = 293 trajectories; DRAQ5, N = 20 cells, *n* = 262 trajectories) **(B)** Percentages of diffusion constant components from multimodal Gaussian fittings for each condition. **(C,D)** Distributions of diffusion constants for individual trajectories in cells treated with Hoechst and DRAQ5, respectively. Green lines represent fitting with triple Gaussian peaks for the entire distribution. Blue and black lines represent single Gaussian portions for slow and fixed diffusions, respectively.

Based on the result of actinomycin D treatment, we tested other DNA intercalators; Hoechst and DRAQ5, which are fluorescent DNA staining dyes usable for live cell imaging. Consistent with the result of actinomycin D treatment, MSD-t plots for Hoechst and DRAQ5 showed a decrease in chromatin motions (Figure 3A).

Results of multiple Gaussian fitting for the distributions of diffusion constants for the intercalators were also in agreement with the result of actinomycin D treatment (Figures 2B,G; Figures 3B–D). *D*_fast_ disappeared, while *D*_fixed_ was drastically increased. Taken together, these results imply that DNA intercalators may increase the rigidity of chromatin so which causes a reduction in the global mobility of chromosomes. Slight differences in portions of *D*_fixed_ and *D*_slow_ for each intercalator may be due to differences in intercalating mechanisms for different intercalators (Almaqwashi et al., 2016a; Almaqwashi et al., 2016b).

### Effects of Cellular Osmotic Pressures on Chromatin Motions

Recent microscopy techniques that interrogate liquid properties of biomolecular condensates have captured subnuclear membraneless organelles that undergo liquid-liquid phase-separation (LLPS) for their formations (Hyman et al., 2014; Banani et al., 2017; Sabari et al., 2020). RNA Polymerase II and essential transcription factors also have been verified to form a cluster with liquid properties, which is so-called a transcriptional condensate (Cho et al., 2018; Sabari et al., 2018).

Especially, the transcriptional condensates and nuclear speckles, which are comparably abundant subnuclear organelles, are known to be functional for gene expression regulation on active genes in the processes of transcription and splicing, respectively. Given that chromatin is where genes are located, those organelles also could be physical constrains for chromatin mobility. The condensates are thought to be formed by cooperative interactions of nucleic acids and proteins that contain intrinsically disordered regions (IDRs). Hydrophobic interaction is considered one of the key driving forces for condensation (Hyman et al., 2014).

**FIGURE 4:**
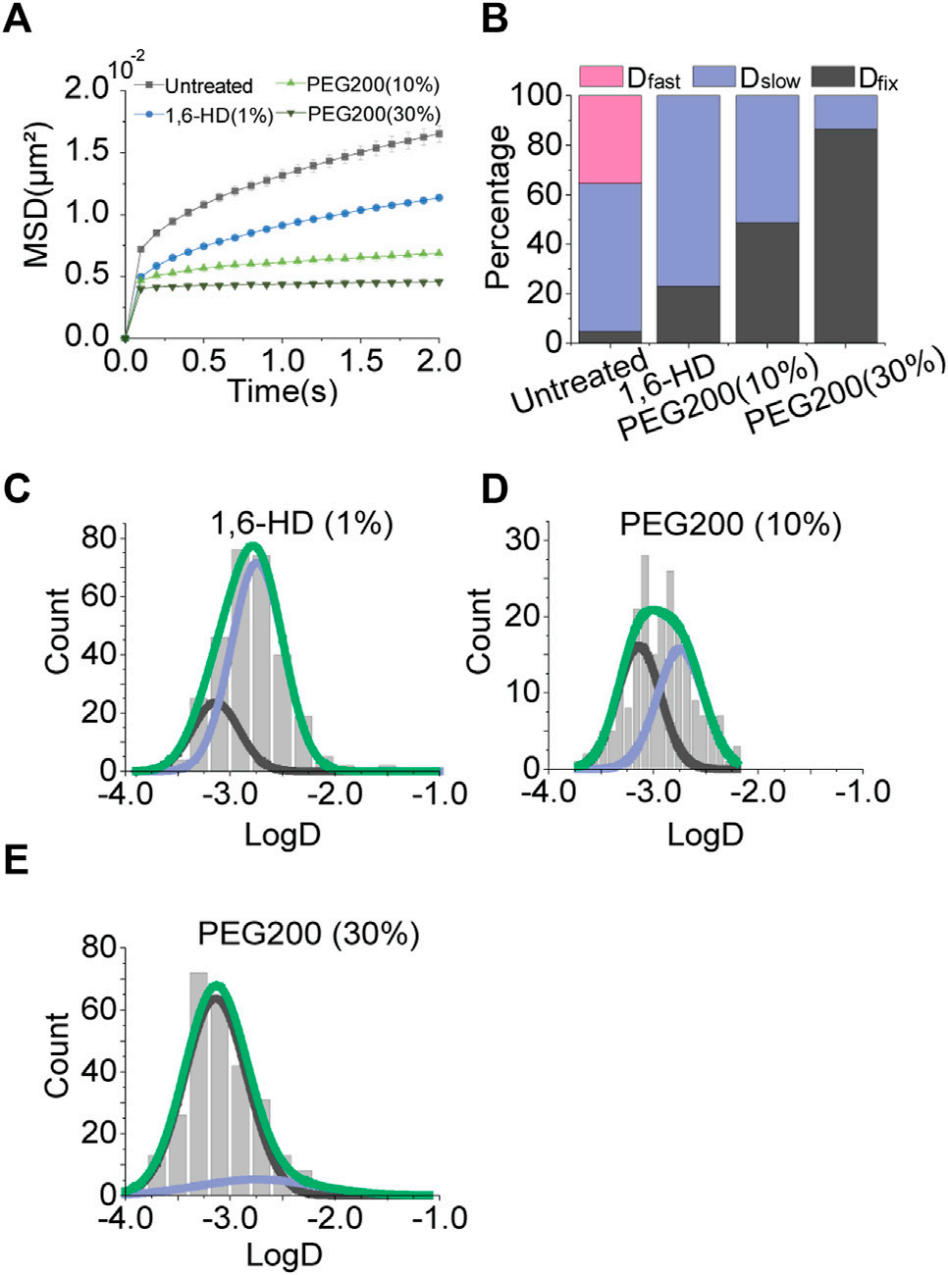
Effects of osmotic pressure. **(A)** Averaged MSD-t plots for each time interval under the normal condition and osmotic pressures (1% 1,6-hexanediol, N = 31 cells, *n* = 314 trajectories; 10% PEG200, N = 20 cells, *n* = 231 trajectories; 30% PEG200, N = 20 cells, *n* = 302 trajectories). **(B)** Percentages of diffusion constant components from multimodal Gaussian fittings for each condition. **(C–E)** Distributions of diffusion constants for individual trajectories in cells treated with 1% 1,6-hexanediol, 10% PEG200, and 30% PEG200, respectively. Green lines represent fitting with triple Gaussian peaks for the entire distribution. Blue and black lines represent single Gaussian portions for slow and fixed diffusions, respectively.

To perturb the condensates, we tested 1,6-hexanediol which is known to disrupt hydrophobic interactions among biomolecules (Cho et al., 2018; Sabari et al., 2018). Although we hypothesized that the disruption of condensates may increase chromatin movements by removing physical constraints, 1,6-hexanediol treatment decreased the chromatin motion (Figure 4A).

With this result, we speculated that osmotic pressure on the cells caused by 1,6-hexanediol treatment may affect chromatin movements. To test this effect, we exposed the cells to hypertonic buffers composed of polyethylene glycol (PEG) molecules. We observed a drastic decrease in chromatin motions proportional to the concentration of PEG (Figure 4A, Supplementary Figure S2). Checking multimodal distributions of diffusion constants, as osmotic pressure increases the portion of *D*_fixed_ becomes dominant (Figures 4B–E). Reduced mobility of chromatin is thought to be due to nuclear volume reduction under osmotic pressure.

### Effects of Chromatin Architectural Proteins on Chromatin Motions

Regarding our previous observation, we hypothesized that increasing nuclear volume may promote chromatin motion by allowing more space for movements. Rather than changing the volume of the nucleus itself, we decided to induce chromatin packing with chromatin architectural proteins, CCCTC-binding factor (CTCF), and Cohesin, which can indirectly increase the nuclear space for the chromatin mobility.

With advanced 3C-based genome-wide sequencing techniques combined with chromatin immunoprecipitation (ChIP), it has been found that CTCF and Cohesin are enriched at the most (not all) of TAD boundaries, indicating that the chromatin loops are formed by those mechanical units (Dekker and Mirny, 2016; Symmons et al., 2016; Nora et al., 2017; Rowley and Corces, 2018). Recent studies with *in vitro* real-time single-molecule assays have shown that CTCF and Cohesin indeed create chromatin loop structures, through the loop-extrusion mechanism. In detail, the ring-shaped Cohesin entraps and extrudes chromatin until it encounters a convergently oriented chromatin-bound CTCF pair (Sanborn et al., 2015; Fudenberg et al., 2016; Ganji et al., 2018). Based on this mechanism, Cohesin extrudes chromatin fibers as a molecular motor, and CTCF determines the boundaries of the loops.

**FIGURE 5:**
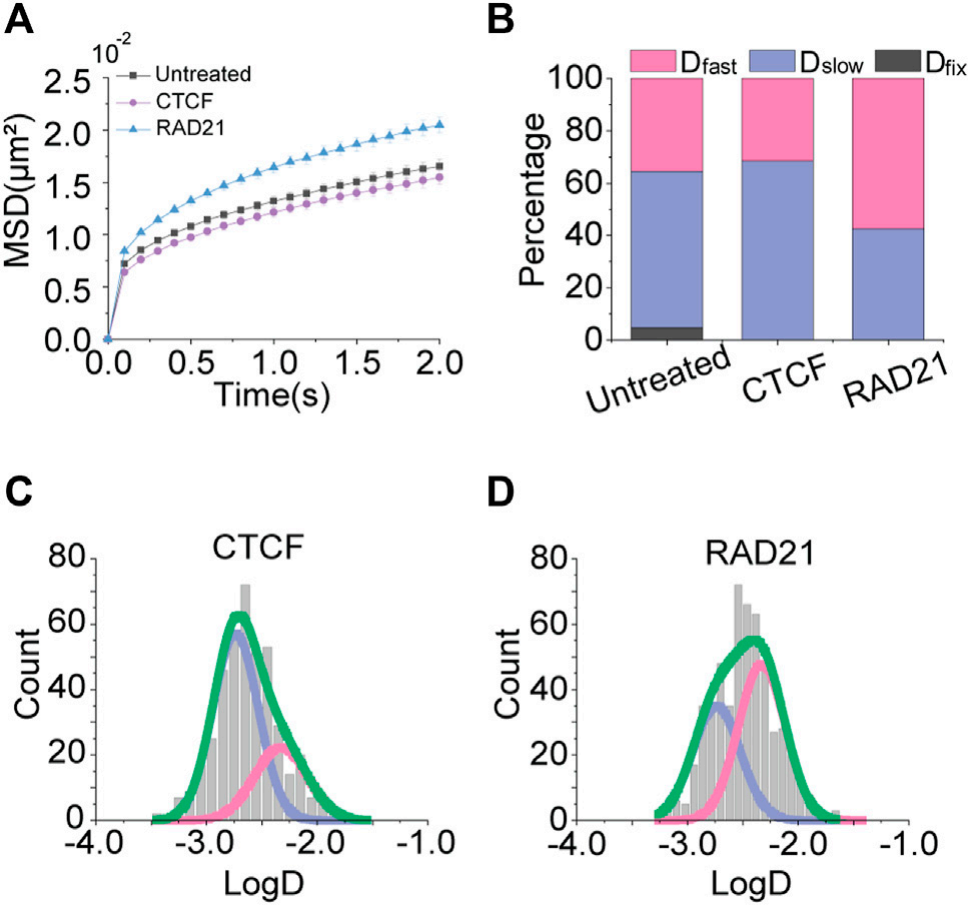
Effects of chromatin architectural protein overexpression. **(A)** Averaged MSD-t plots for each time interval under the normal condition and with overexpression of CTCF and RAD21. (CTCF, N = 32 cells, *n* = 449 trajectories; RAD21, N = 45 cells, *n* = 566 trajectories) **(B)** Percentages of diffusion constant components from multimodal Gaussian fittings for each condition. **(C,D)** Distributions of diffusion constants for individual trajectories in cells treated with CTCF and RAD21 overexpression, respectively. Green lines represent fitting with triple Gaussian peaks for the entire distribution. Pink and blue lines represent single Gaussian portions for fast and slow diffusions, respectively.

To test the effects of chromatin architectural proteins on chromatin movements, we overexpressed CTCF and RAD21, a subunit of the Cohesin complex, independently in the cells. As we expected, CTCF did not show a significant change in the mobility of chromatin, meaning that the boundary element CTCF itself does not influence the chromatin packing (Figure 5A). On the contrary, the overexpression of RAD21 increased chromatin mobility, suggesting that Cohesin mainly causes chromatin loop formations that lead to chromatin packing. As a result, the effective volume for the chromatin movement is increased as if the nuclear space is widened.

The multimodal distributions of diffusion constants also showed the aspect (Figures 5A–C). The portion of *D*_fast_ has increased for the overexpression of RAD21, indicating that the long-range movement of chromatin becomes more frequent.

We further performed knockdown experiments on CTCF and Rad21. As expected, knockdown of CTCF did not significantly alter chromatin mobility. However, the knockdown of Rad21 still increased chromatin mobility. We speculate this result might be due to that knockdown of Rad21 seems to have a greater effect on reducing rigidity and making chromatin more flexible than the effect of reducing effective volume, similar to the results of transcription inhibitors (Supplementary Figure S4).

## Discussion

**FIGURE 6:**
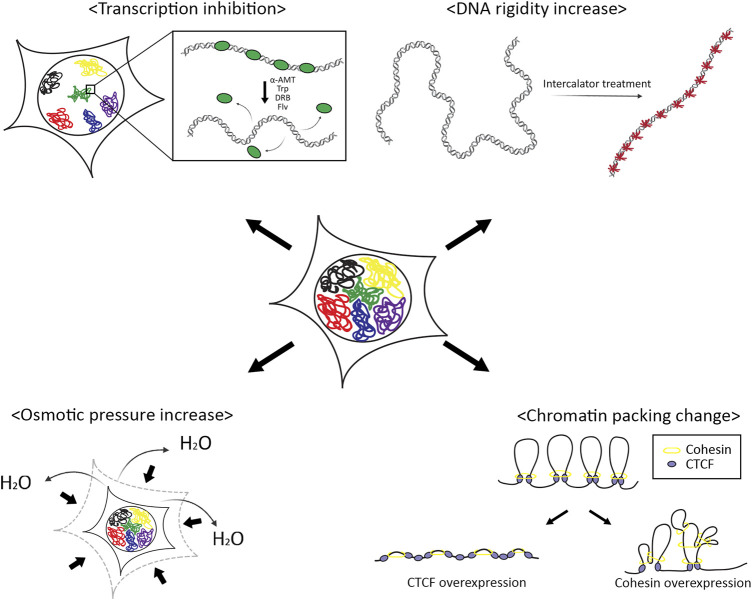
Schematic summary of effects of physical perturbations on chromosomes. The movement of a chromosome is affected by the physical properties of chromatin fibers, rigidity, and volume confinement. Transcription factors associated with chromatin and DNA intercalators make chromatin more rigid. Osmotic pressure and chromatin compaction by chromatin architectural proteins reduce effective nuclear space for chromosome movements.

The genetic material, DNA which is wrapped into chromatin fibers, is a physical entity in a finite space, the nucleus. The physical properties of chromatin, therefore, are linked to gene expression regulation (Figure 6). We investigated how physical perturbations alter chromatin dynamics in this study.

We tested various chemicals that can inhibit transcription or that can intercalate into the DNA double helical structure inducing increased chromatin stiffness. We observed that transcription inhibitors increased chromatin mobility while DNA intercalators drastically reduced chromatin motions. These opposite responses of the chromatin mobility to different chemicals may indicate the same physical consequence; transcription machinery affected by the transcription inhibitors apply physical constraints by forming chromatin loops and repressing the movements of chromatin. Thus, inhibiting transcription should induce increased flexibility in chromatin structures. On the other hand, DNA intercalators stuck between bases in DNA make the chromatin more rigid.

Upon the treatment of osmotic agents to the cells, we found the declined motions of chromatin. Osmotic pressure with a hypertonic solution to the cell causes an outward flow of water molecules across the nuclear membrane, with shrinking volume to adjust concentrations in and out of the nucleus (Finan and Guilak, 2010). The reduced chromatin mobility could be a result of the volumetric effect or increased viscosity in the nucleus. We further tested whether inducing chromatin packing shows the same result. With the overexpression of chromatin architectural proteins, CTCF and RAD21, we observed increased chromatin mobility with RAD21 overexpression, but not with CTCF. These results are inconsistent with the molecular functions of each protein. During chromatin loop formation, the cohesion complex creates loops through the loop extrusion mechanism, and CTCF acts as anchors of the loops. Thus, CTCF cannot directly influence the chromatin loop formation while cohesion can promote more chromatin loop formation which results in the compaction of chromatin volume, which gives more spaces for chromatin motions in the nucleus.

Taken together, our experiments perturb the physical properties of chromosomes and showed alterations in the physical environment directly influence the chromosome motions, which is also related to transcriptional activities. Since we measured the motions of chromosomes with telomere regions, which may not fully represent chromosome movements, additional measurements from the movements of whole chromosomes should be required to confirm our conclusions in a further study.

## Methods

### Cell Culture

The human cervical cancer cell line HeLa (Korean Cell Line Bank # 10002) was cultured in Dulbecco’s modified Eagle medium (DMEM) with high glucose, devoid of phenol red (Gibco, #31053028), supplemented with 10% fetal bovine serum (FBS) (Gibco, #12483020), 1% antibiotic-antimycotic (Gibco, #15240062), 1% GlutaMAX™ supplement (Gibco, #35050061), and 1% sodium pyruvate (Gibco, #11360070). Cells were cultured at 37°C and 5% CO2 in a humidified incubator. Cell lines were periodically tested for *Mycoplasma* contamination using the e-MycoTM *Mycoplasma* PCR Detection kit (iNtRON).

### Fluorescence Imaging With Telomere Labeling

For live cell imaging, cells were imaged in DMEM with high glucose, devoid of phenol red, supplemented with 10% FBS and 1% antibiotic-antimycotic. Before imaging, cells were cultured in 35-mm glass-bottom dishes (SPL-confocal dish, #100350) until 80% confluency was reached. For telomere labeling, cells were transfected with 300 ng of SpydCas9-3XmCherry-SgRNA/Telomere-All-in-one vector (Addgene, #85717) and 1,500 ng of SpySgRNA/DTS13-Telomere (Addgene, #85715) using FuGENE^®^ HD Transfection Reagent (Promega, #E2311) in a ratio of 1:5 (Pawluk et al., 2016). Cells were imaged at 37°C and 5% CO2 in a microscope stage-top incubator.

For fixed cell imaging, the culture medium was exchanged with 4% paraformaldehyde (PFA) (Biosensang,#PC2031-100-00) after washing with Dulbecco’s phosphate buffered saline (DPBS) (WELGENE, #LB001-02). After 10 min incubation at room temperature, PFA was exchanged with 1x PBS. To completely remove PFA, cells were incubated in DPBS for 5 min, and this process was repeated three times.

### Transcription Inhibitor Treatment

Cells were cultured in 35-mm glass-bottom dishes before treatment with transcription inhibitors. For each transcription inhibitor, cells were incubated for 2 h under actinomycin D (Sigma-Aldrich, #A1410), 100 μM 5,6-Dichloro-1-β-D-ribofuranosylbenzimidazole (DRB) (Sigma-Aldrich, #D1916), 5 μM α-amanitin (Sigma-Aldrich, #23109-05-9), 10 μM flavopiridol (Sigma-Aldrich, #F3055), and 125 nM triptolide (Sigma-Aldrich, #T3652) diluted in dimethyl sulfoxide (Sigma-Aldrich, #D8418), respectively, before imaging.

### DNA Intercalator Treatment

Cells were cultured in 35-mm glass-bottom dishes before treatment of DNA intercalators. For each DNA intercalator, cells were incubated for 2 h under 2 μM actinomycin D (Sigma-Aldrich, #A1410), for 10 min under 5 μM DRAQ5 (Abcam, #ab108410) and 9 μM Hoechst (Thermo Fisher Scientific, #62249).

### Osmotic Agent Treatment

Cells were cultured in 35-mm glass-bottom dishes before treatment with osmotic agents. Cells were treated with 1% (wt/vol) 1,6-hexanediol (Sigma-Aldrich, #240117) for 5 min, with 10%, 30% (wt/vol) Polyethylene glycol (PEG200) (Sigma-Aldrich, #P3015) for 5 min, before imaging on the microscope stage.

### Overexpression of Chromatin Architectural Proteins

For expression of chromatin architectural proteins, CTCF or RAD21 (a subunit of cohesion complex), 1 µg of pKS070-pCAGGS-3XFLAG-(human)CTCF-eGFP (Addgene, #156448), and 1 µg of Dendra2-Rad21-C-18 (Addgene, #57737) expression vectors were transfected to the cells for each experiment. We confirmed the expression of each protein in cell nuclei, with GFP signals for CTCF and Dendra2 signals for RAD21, respectively. We imaged and collected data from fluorescence positive cells.

### Microscopy With Highly Inclined and Laminated Optical Illumination

Cell imaging was performed with highly inclined and laminated optical (HILO) illumination from a Nikon TIRF microscope based on the Nikon Eclipse Ti microscope body. For detections of telomere loci, mCherry was excited with a built-in 561 nm laser in the microscope. Images were acquired through a 100X objective lens of 1.49 numerical aperture (Nikon, Plan Apochromat TIRF 100x oil-type) with the electron multiplying charge coupled device (EMCCD) (Andor, iXon Ultra 897), and processed using the NIS-Elements software (Nikon). For streaming image acquisition, snapshots were taken every 100 ms for 2 min. Image analysis was performed using ImageJ scripts and custom-built MATLAB codes for mean square displacement calculation.

### Single Locus Tracking

Image analysis was performed using ImageJ scripts and custom-built MATLAB codes for mean square displacement calculation. Single locus tracking was performed by an ImageJ plugin-in software, TrackMate. Each fluorescent locus per frame was identified by Laplacian Gaussian fitting with an LoG detector in the TrackMate. Background noise was minimized by the LoG detector which is based on applying an LoG filter on each image and detecting local maxima. 2D trajectories of X-Y coordinates over time for each particle were obtained.

### MSD Calculation

For telomere movement analysis, single particle analysis was performed using a custom-built MATLAB code. MSD equation was used to measure the characteristic of diffusion with time-dependent trajectory information of each particle. The mean square displacement (MSD) was obtained through this formula if we know the X-Y coordinates and time interval in 2D for each frame in the trajectory we obtained and the number of total frames. We calculated MSD-values for each individual track using a method previously reported (Qian et al., 1991; Saxton, 1997) where MSD (nΔt) for a given time lag (nΔt) is defined as an average over all points for the given time lag,

$$\mathrm{MSD}\left(n\Delta t\right)=\frac{1}{N-n}\sum_{i=1}^{N-n}\left[x\left(i+n\right)-x{\left(i\right)}^{2}\right]+{\left[y\left(i+n\right)-y\left(i\right)\right]}^{2},$$

where N represents the length of trajectory and Δt the time delay between frames (Weimann et al., 2013).

Plotting MSD-time curve was performed using the custom-built MATLAB code. The MSD of particles tracked for more than 20 s were collected and averaged over time to obtain an ensemble MSD graph. For estimation of a diffusion coefficient (*D*) and an anomalous diffusion parameter (*α*), MSD-t plots were fitted by 4*Dt* ^α^, for two-dimensional motions.

## Data Availability

The original contributions presented in the study are included in the article/Supplementary Material; further inquiries can be directed to the corresponding authors.
