# Supplementary material for: Effects of Transcription-Dependent Physical Perturbations on the Chromosome Dynamics in Living Cells
Source: Front Cell Dev Biol. 2022 Jul 7;10:822026. doi: 10.3389/fcell.2022.822026 (PMC9302598; doi:10.3389/fcell.2022.822026)

**SUPPLEMENTARY INFORMATION**

**
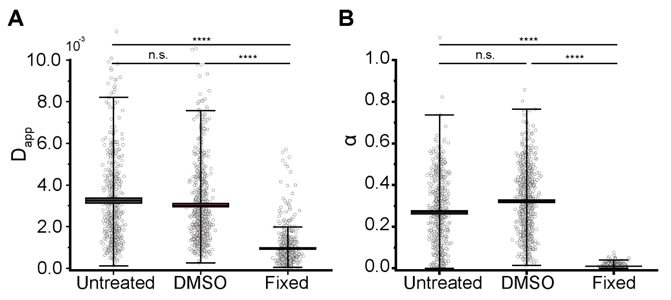
**

**Supplementary Figure 1. Distributions of apparent diffusion constants and anomalous coefficients from individual single chromatin locus trajectories.**

Distributions of (**A**) diffusion constants and (**B**) anomalous coefficients from individual single chromatin locus trajectories, for normally cultured cells, DMSO treated cells, and fixed cells. Error bars show mean ± standard errors (s.e.). *, **, ***, **** indicate statistical significance at p < 0.10, p < 0.05, p < 0.01 and p < 0.001, respectively, with unpaired two-tailed Student’s t-tests.


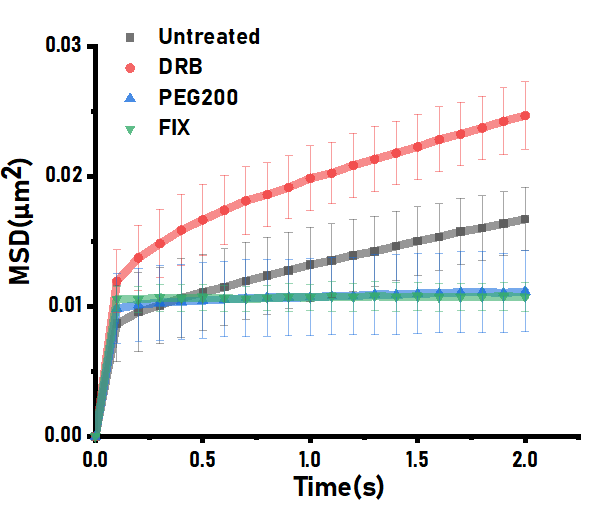


**Supplementary Figure 2. Effects of transcription inhibition and osmotic pressure on chromosome movement in HEK293 cells.**

The effects of transcriptional perturbation and osmotic pressure in Hek293 cells were consistent with those in Hela cells. Averaged MSD-t plots for each time interval in HEK293T cells under normal culture media (N = 24 cells, n = 181 trajectories), DRB treatment (N = 13 cells, n = 84 trajectories), PEG200 treatment (N = 13 cells, n = 288 trajectories) and fixed cells (N = 23 cells, n = 147 trajectories).


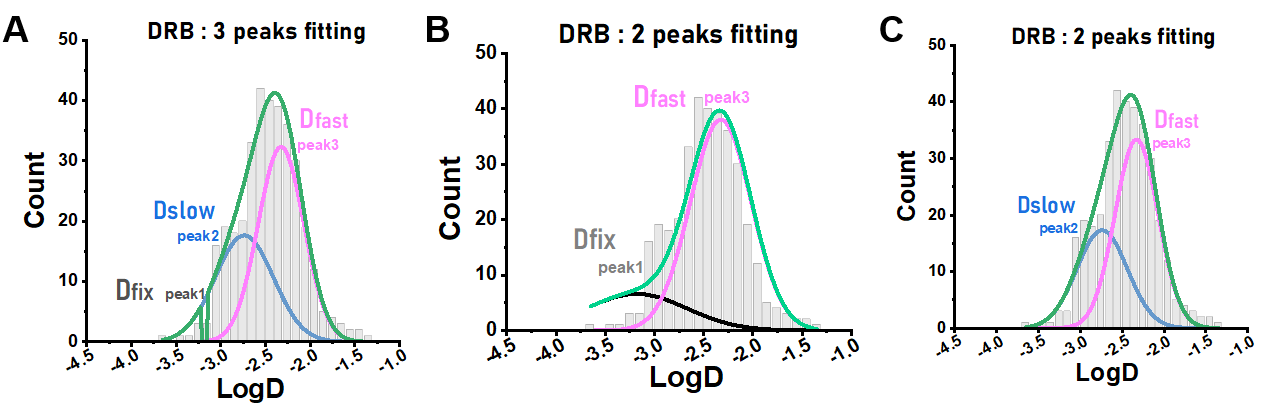


**Supplementary Figure 3. Procedure for multimodal Gaussian fitting with an example of diffusion constant distribution from DRB treat cells.**

For our diffusion portion analysis with multiple Gaussian peaks, we firstly tried to fit each distribution of diffusion constants with triple Gaussian peaks, *D*_fix_, *D*_slow_, and *D*_fast_, using the Origin software (**Table S2**). In a result, if any of the following conditions is met, it is considered as a fitting failure: 1) Fitting cannot be done within 100 iterations. 2) The fitted parameters, such as area and FWHM in the Gaussian model, are improbable. 3) An adjusted R-Square value is less than 0.9. If triple Gaussian fits are successful we obtained portions of each diffusion component. If one of triple Gaussian fits is failed in a result, we fitted again the distribution with double Gaussian peaks without the failed component. For example, in the distribution of diffusion constants for DRB treated cells, (**A**) We firstly tried to fit a distribution with triple Gaussian peaks. But the area of Gaussian of *D*_fix_ was less than zero, we regard it as a fitting failure. The area of Gaussian for *D*_fix_ was -47.3 by Origin software calculation. (**B**) In case of double Gaussian fitting with *D*_fix_ and *D*_fast_, the adjacent R-square value was 0.869 and the width (FWHM) of *D*_fix_ seemed as a failed fitting. (**C**) Fitting with *D*_slow_ and *D*_fast_ met our criteria for successful fitting.


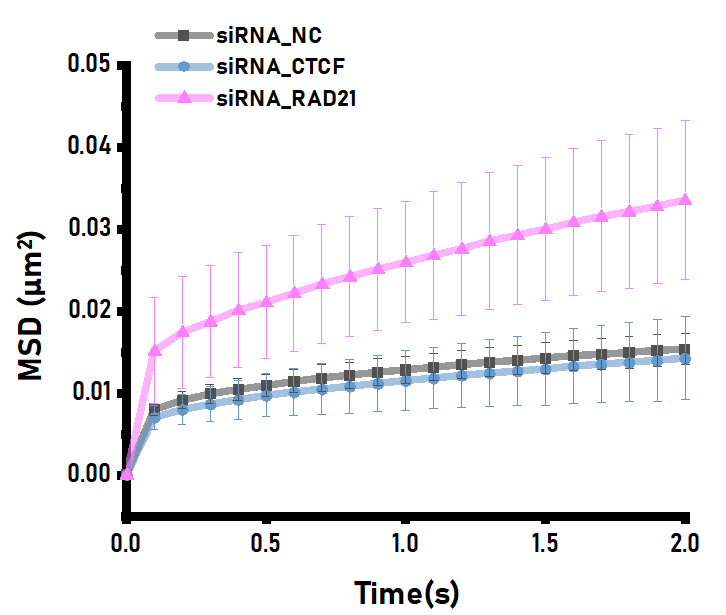


**Supplementary Figure 4. Knockdown experiments of CTCF and Rad21 via Transfection of siRNA.**

Averaged MSD-t plots for each time interval in negative control cells (N = 21 cells, n = 160 trajectories), CTCF-knockdown cells (N = 19 cells, n = 197 trajectories), and RAD21- knockdown cells (N = 20 cells, n = 137 trajectories). For RNA interference experiments, telomere-labeled cells were transfected with siRNA using Lipofectamine RNAiMAX (Invitrogen # 13778150). After the incubation for 48 hours, we changed the medium with DMEM without FBS to a fresh one. Then, the transfected cells were imaged. When performing imaging, we used fluorescein-labeled siRNA to select only transfected cells. The used siRNAs were: CTCF (sense: 5’-ACAAGCUUGACCAUACCAGtt-3’, antisense: 5’-CUGGUAUGGUCAAGCUUGUtt-3’, Bioneer) and RAS21 (sense: 5’-UACUGAUGGAAAGAAGUGUtt-3’, antisense: 5’-ACACUUCUUUCCAUCAGUAtt-3’, Bioneer). As a negative control, Fluorescein-labeled Negative Control Sence (Bioneer #SN-1022) was used.

**Table S1. Diffusion constants and anomalous coefficients from anomalous diffusion fitting.**


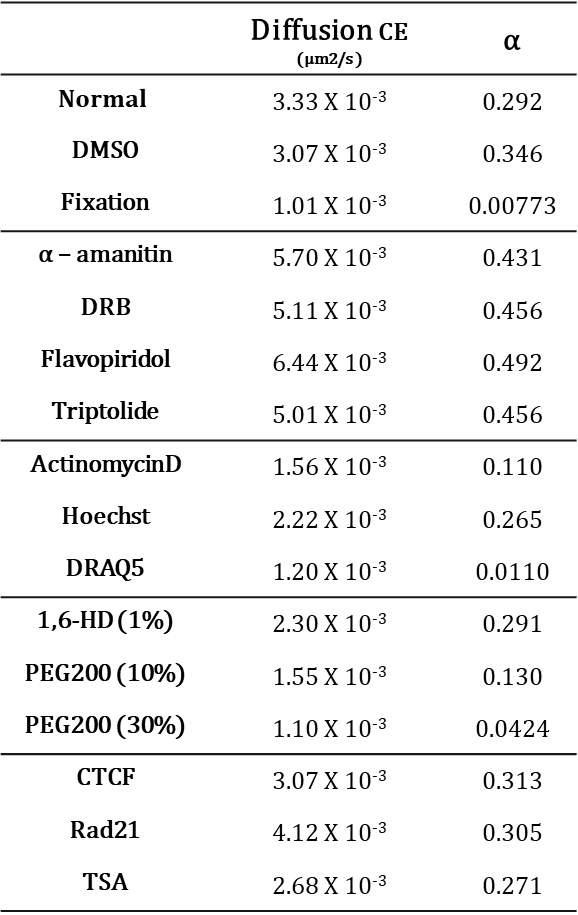


**Table S2. Parameters for multimodal Gaussian fitting.**


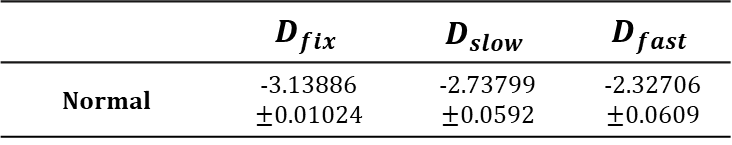

Supplement: Supplementary file 1 [file DataSheet1.docx]
